# Role of forest edges and other seminatural linear landscape features in structuring wild bee habitat connectivity in intensively managed landscapes

**DOI:** 10.1111/cobi.70152

**Published:** 2025-09-30

**Authors:** Markus A. K. Sydenham, Anders Nielsen, Yoko L. Dupont, Claus Rasmussen, Henning B. Madsen, Marianne S. Torvanger, Bastiaan Star

**Affiliations:** ^1^ The Norwegian Institute for Nature Research Oslo Norway; ^2^ Department of Landscape and Biodiversity Norwegian Institute of Bioeconomy Research (NIBIO) Ås Norway; ^3^ Department of Agroecology Aarhus University Aarhus Denmark; ^4^ Department of Biology University of Copenhagen Copenhagen Denmark; ^5^ Centre for Ecological and Evolutionary Synthesis, Department of Biosciences University of Oslo Oslo Norway

**Keywords:** bees, connectivity, conservation planning, grassland, pollinators, restoration, abejas, conectividad, pastizales, planeación de la conservación, restauración, 蜜蜂, 连通性, 保护规划, 传粉者, 恢复, 草原

## Abstract

Pollinator conservation schemes typically focus on conserving existing, restoring degraded, or creating new wild bee habitats. Their effectiveness depends on dispersal corridors enabling habitat colonization by bees. However, the role of seminatural linear landscape structures (LLS) in connecting pollinator communities across intensively managed landscapes remains poorly understood. We analyzed 953 occurrences of wild bees comprising 79 nonparasitic species sampled at 68 study sites across a Norwegian and a Danish landscape. We first tested whether bee species richness was positively associated with the lengths of seminatural LLS in bee foraging ranges of study sites while controlling for local plant species richness. We then combined maps identifying seminatural LLS with least‐cost path (LCP) analysis to determine whether bee species compositional similarity, a proxy for connectivity, decreased as LCP length increased. The length of seminatural LLS, such as forest edges, was positively correlated with bee species richness and habitat connectivity. Specifically, wild bee species richness sampled along roadsides increased as the length of seminatural LLS increased in 1.5 km circles around the study sites, and increased as local plant species richness increased. The most likely dispersal routes between our bee communities tracked forest edges. The length of LCPs provided better models of bee species compositional similarity than geographic distance, suggesting that seminatural LLS, particularly forest edges, act as dispersal corridors in intensively managed landscapes. However, bee species compositional similarity among communities depended on site‐specific plant species richness and similarity in plant community composition, which highlights the importance of improving the habitat quality of seminatural LLS if they are to function as dispersal corridors. Our findings suggest that maps of LCPs can be used to identify important dispersal corridors between bee habitats and to direct wild bee habitat management actions along seminatural LLS to facilitate the dispersal of bees in intensively managed landscapes.

## Abstract (es)

El papel de los bordes forestales y otros rasgos lineales seminaturales del paisaje en la estructuración de la conectividad de hábitats de abejas silvestres en paisajes con manejo intensivo

**Resumen:**

Los programas de conservación de polinizadores suelen centrarse en conservar los hábitats existentes, restaurar los degradados o crear nuevos hábitats para las abejas silvestres. Su eficacia depende de los corredores de dispersión que permiten la colonización del hábitat por parte de las abejas. Sin embargo, todavía se sabe poco sobre el papel de las estructuras paisajísticas lineales seminaturales (LLS) en la conexión de las comunidades de polinizadores a través de paisajes con gestión intensiva. Analizamos 953 ejemplares de abejas silvestres, que pertenecientes a 79 especies no parasitarias, recolectados en 68 sitios de estudio en un paisaje noruego y danés. En primer lugar, comprobamos si la riqueza de especies de abejas estaba asociada positivamente con la longitud de las LLS seminaturales en las zonas de alimentación de las abejas de los sitios de estudio, controlando al mismo tiempo la riqueza de especies vegetales locales. A continuación, combinamos mapas que identificaban las LLS seminaturales con un análisis de la ruta de menor costo (LCP) para determinar si la similitud en la composición de las especies de abejas, un indicador de la conectividad, disminuía a medida que aumentaba la longitud de la LCP. La longitud de las LLS seminaturales, como los bordes de los bosques, se correlacionó positivamente con la riqueza de especies de abejas y la conectividad del hábitat. En concreto, la riqueza de especies de abejas silvestres muestreadas a lo largo de los bordes de las carreteras aumentó a medida que incrementaba la longitud de los LLS seminaturales en 1.5 km de los sitios de estudio e incrementó a medida que aumentaba la riqueza de especies vegetales locales. Las rutas de dispersión más probables entre nuestras comunidades de abejas seguían los bordes de los bosques. La longitud de los LCP proporcionó mejores modelos de similitud en la composición de las especies de abejas que la distancia geográfica, lo que sugiere que los LLS seminaturales, en particular los bordes de los bosques, actúan como corredores de dispersión en paisajes con gestión intensiva. Sin embargo, la similitud en la composición de las especies de abejas entre las comunidades dependía de la riqueza de especies vegetales específicas del sitio y de la similitud en la composición de la comunidad vegetal, lo que destaca la importancia de mejorar la calidad del hábitat de los LLS seminaturales si queremos que funcionen como corredores de dispersión. Nuestros hallazgos sugieren que los mapas de rutas de menor costo pueden utilizarse para identificar corredores de dispersión importantes entre los hábitats de las abejas y para orientar las acciones de gestión del hábitat de las abejas silvestres a lo largo de estructuras paisajísticas lineales seminaturales, con el fin de facilitar la dispersión de las abejas en paisajes con gestión intensiva.

## 森林边缘及其他半自然线性景观特征帮助在密集管理景观中提高野生蜜蜂栖息地连通性

**【摘要】** 传粉者保护计划通常聚焦于保护现有的野生蜜蜂栖息地, 或是恢复退化的及建立新的野生蜜蜂栖息地。这些计划的有效性取决于其能否为蜜蜂栖息地的建立提供扩散廊道。然而, 目前尚不清楚半自然线性景观结构(seminatural linear landscape structure, LLS)在密集管理景观中帮助传粉者群落连通的作用。本研究分析了挪威及丹麦景观68个研究地点记录的953次野生蜜蜂活动, 包括了79种非寄生物种。我们首先检验了在控制当地植物物种丰富度的情况下, 蜜蜂物种丰富度是否与研究地点蜜蜂觅食范围内半自然LLS的长度呈正相关。随后, 我们结合识别半自然LLS的地图与最小成本路径(least‐cost path, LCP)分析, 确定了蜜蜂物种组成相似性(代表连通性)是否会随LCP长度增加而下降。结果显示, 半自然LLS(如森林边缘)的长度与蜜蜂物种丰富度和栖息地连通性呈正相关。具体而言, 沿道路边采样的野生蜜蜂物种丰富度会随着研究地点1.5公里范围内半自然LLS长度增加而增加, 也会随着当地植物物种丰富度增加而增加。蜜蜂群落之间最可能的扩散路线是沿森林边缘。与地理距离相比, LCP的长度能更好地模拟蜜蜂物种组成的相似性, 这表明半自然LLS, 尤其是森林边缘, 在密集管理的景观中起到了扩散廊道的作用。然而, 群落间蜜蜂物种组成的相似性取决于地点特异的植物物种丰富度和植物群落组成相似性, 这强调了改进半自然LLS栖息地质量的重要性, 从而帮助其发挥扩散廊道的作用。我们的研究结果表明, LCP地图可用来识别蜜蜂栖息地之间的重要扩散廊道, 并指导沿半自然LLS的野生蜜蜂栖息地管理行动, 以促进蜜蜂在密集管理景观中的扩散。**【翻译:胡怡思;审校:聂永刚】**

## INTRODUCTION

Land‐use intensity and the associated loss of seminatural habitats are the main drivers of pollinator declines and threaten ecosystem functioning in cultural landscapes (Dicks et al., 2021). Pollinator diversity increases as habitat size (Blaauw & Isaacs, 2014; Steffan‐Dewenter et al., 2006) and quality increase (Rollin et al., 2019). Conserving or restoring habitats is a central component of many pollinator conservation schemes (Senapathi et al., 2017). However, species composition in habitats is dynamic (Leibold et al., 2004). Species disperse across the landscape and colonize patches of habitat (Franzén & Nilsson, 2010), where they may remain or become extirpated before the patch is potentially colonized again (Hanski, 1998; Vellend, 2010). Spatial connectedness between pollinator habitat patches (Beduschi et al., 2018) and land‐use history—or temporal connectedness—(Griffin et al., 2017) are important contributors to species composition (Taylor et al., 1993). Mitigating insect declines and preserving ecological functions, such as pollination, therefore, require an understanding of how land‐use management and changes in habitat availability affect dispersal rates and connectedness of pollinator communities across landscapes (Cranmer et al., 2012; Vasiliev & Greenwood, 2023).

Pollinator communities often appear to display some degree of dispersal limitation such that species compositional similarity decreases as geographic distance between communities increases (Carstensen et al., 2014; Trøjelsgaard et al., 2015; Vitali et al., 2024) and communities become increasingly dominated by mobile species as habitat connectivity is reduced (Marini et al., 2014). Also, the occurrence of species in habitats decreases as geographic distance to the nearest occurrence of that species increases (Sydenham et al., 2017). Although distance−decay relationships in species compositional similarity are indicative of ecological communities being dispersal‐limited (Soininen et al., 2007), they do not provide explicit tests of how landscape conditions affect connectivity. For instance, pollinator communities separated by the same distance but with higher proportions of habitat between them tend to be more similar than expected from the distance−decay curve, whereas communities separated by unfavorable land‐use types are less similar (Beduschi et al., 2018). An alternative distance measure to geographic distance is the least‐cost path (LCP) length, which shows the route between 2 communities where the presence of habitats reduces resistance to movement and thereby reflects the most likely dispersal route (Adriaensen et al., 2003; Etherington, 2016; Zeller et al., 2012). In ant communities, resistance to movement is a better predictor of species turnover than geographic distance (Liu et al., 2018). However, for pollinators in intensively managed landscapes, it remains uncertain whether the presence of specific habitat features can reliably indicate likely dispersal corridors. Addressing this gap requires identifying landscape features that provide habitat for pollinators and assessing their role in facilitating dispersal and connectivity across fragmented landscapes.

The species richness and abundance of pollinators that a landscape can sustain depend on the availability and size of habitat (Krauss et al., 2009; Steffan‐Dewenter et al., 2006). Seminatural linear landscape structures (LLS) along unfavorable land‐use types, such as urban areas, closed forests, intensively managed grasslands, and cereal fields, provide important resources for pollinators in fragmented landscapes (Eldegard et al., 2015; Johansen et al., 2022; Sydenham et al., 2023; von Königslöw, 2021). Pollinators can forage or nest in seminatural LLS, such as forest‐field edges (Kells & Goulson, 2003; Sõber et al., 2020; Sydenham et al., 2014), forest‐shrubland edges (Glenny et al., 2023), grassland edges (Cole et al., 2015), road verges (Hopwood, 2008), and edges around sparsely vegetated areas, such as quarries and other areas of anthropogenic influence (Heneberg & Bogusch, 2020). Moreover, improving or introducing edge habitats in the form of flower strips (Haaland et al., 2011; von Königslöw et al., 2021) or hedgerows (Morandin & Kremen, 2013) can provide pollinators with resources that are otherwise limited in intensively managed, and consequently, fragmented landscapes.

Wild bees are efficient pollinators of many plants (Willmer et al., 2017), but compared with more mobile insects, they may be more vulnerable to habitat isolation (Jauker et al., 2009) because they are central place foragers. Most species have home ranges of <1 km; smaller species have shorter ranges (Greenleaf et al., 2007; Zurbuchen et al., 2010). Even large bees often show limited movement between habitat patches (Franzén et al., 2009), and bee metapopulation dynamics can be highly variable in terms of extinction and colonization events (Franzén & Nilsson, 2010). There is some evidence that bee communities in intensively managed landscapes can be dispersal‐limited (Beduschi et al., 2018; Sydenham et al., 2017) and that the presence of open linear corridors in forested landscapes can increase the colonization rate of solitary bees (Griffin & Haddad, 2021). By providing bees with nesting and foraging sites (Cole et al., 2017; Kells & Goulson, 2003; Morandin & Kremen, 2013; Osborne et al., 2008) and by guiding their flight direction (Cranmer et al., 2012), the presence of seminatural LLS may function as dispersal corridors and increase the dispersal rate of species through the landscape. Conversely, habitat loss and fragmentation can affect habitat connectivity if the availability of seminatural habitat LLS is reduced along dispersal corridors.

The density of seminatural LLS is positively associated with pollinator abundance and diversity (Priyadarshana et al., 2024) and can facilitate pollinator foraging, as evidenced by increased crop pollination (Hass et al., 2018; Martin et al., 2019). By providing habitat resources and enhancing foraging efficiency, seminatural LLS may act as steppingstones along dispersal routes (Menz et al., 2011), thereby supporting metapopulation dynamics (Hanski, 1998; Saura et al., 2014). Although the movement patterns of bees related to foraging and dispersal are distinct, they share certain commonalities. For instance, large‐bodied bees, such as bumblebees, are capable of both foraging (Greenleaf et al., 2007) and dispersing over greater distances (López‐Uribe et al., 2019) than smaller‐bodied species, such as most solitary bees, which tend to respond more strongly to land‐use intensity and operate at smaller spatial scales (Steffan‐Dewenter et al., 2002). Previous studies have explored the contributions of seminatural LLS to enhance local pollinator diversity and foraging movement, but the potential role of seminatural LLS in enhancing wild bee habitat connectivity in fragmented landscapes—and whether this role differs between bumblebees and solitary bees—has, to our knowledge, not yet been formally investigated.

**FIGURE 1:**
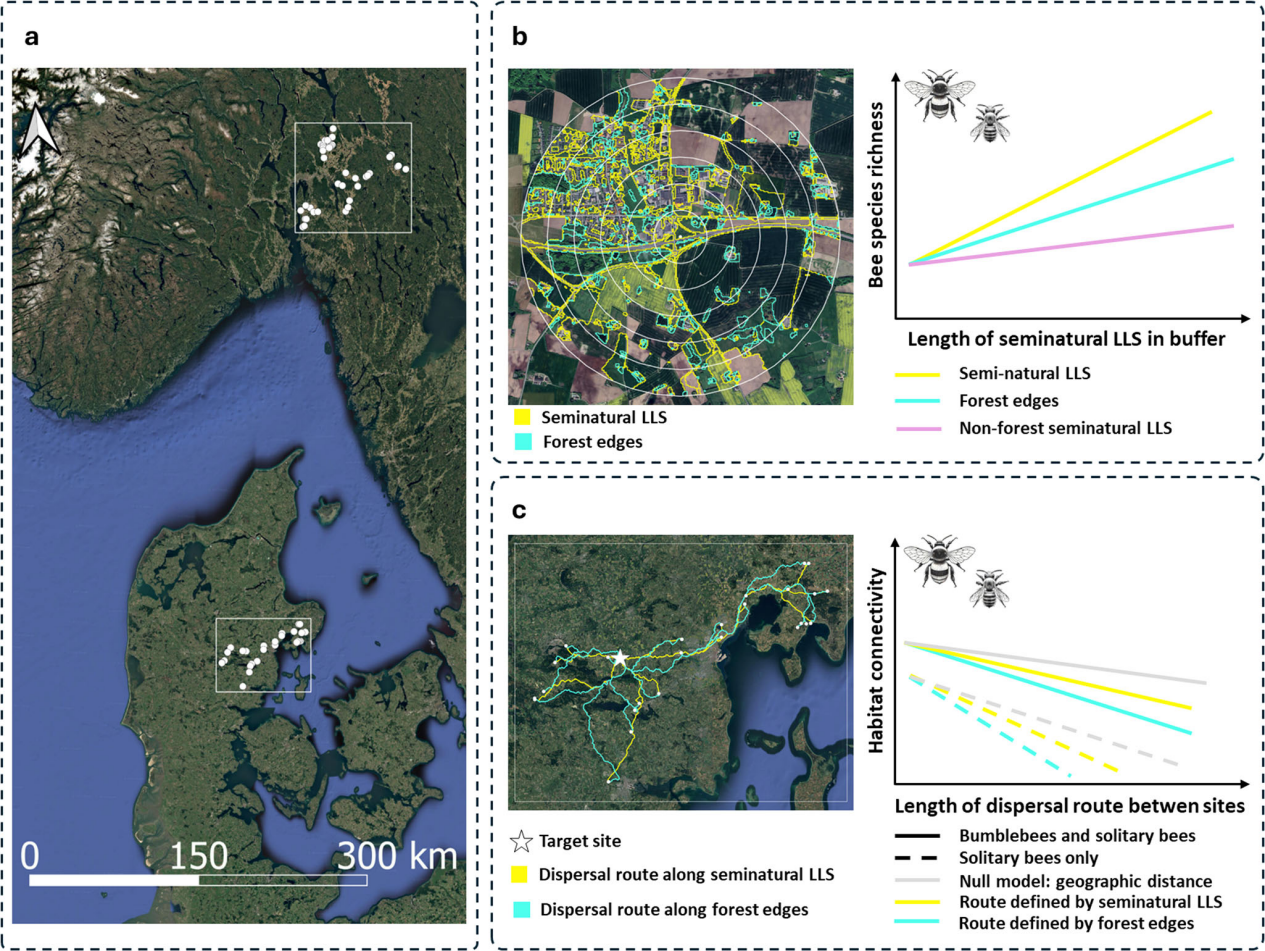
For wild bee communities in Denmark and Norway, (a) the 68 roadside and linear habitats sampled, (b) bee species richness relative to length of seminatural linear landscape structures (LLSs) seminatural, and (c) habitat connectivity relative to length of dispersal corridors for wild bees. Map data from Google (2024) via QGIS (2024).

We addressed these gaps by analyzing data from Sydenham et al. (2024), for which 68 wild bee communities were sampled across a landscape in Norway and a landscape in Denmark. We tested 2 hypotheses (Figure 1): seminatural LLS provide habitat resources for wild bees so that bee species richness increases as the length of seminatural LLS increases in the foraging ranges of bees, and seminatural LLS provide dispersal corridors for wild bees, such that bee species compositional similarity is more strongly related to hypothesized dispersal routes that track seminatural LLS than to geographic distance. We also assessed whether these contributions differ between bumblebees and solitary bees. By assessing the role of seminatural LLS as habitat for wild bees and identifying likely dispersal corridors, we sought to provide guidance for conservation planning by identifying priority sites along dispersal corridors for habitat improvement schemes.

## METHODS

### Sampling

We used data from Sydenham et al. (2024). These authors recorded plant−bee interactions in 68 study sites in 2021 (Figure 1): 27 sites in Denmark and 41 sites in Norway. Each site consisted of a 50 × 2 m transect placed along roadside vegetation. Sites were selected in 2020 based on the criteria that sites should be 1 km apart and contain bee‐visited plants in at least one of the following plant families: Asteraceae, Rosaceae, Ranunculaceae, and Fabaceae. Sites were sampled once in May, June, and July, which covers the main flowering period. To standardize sampling times across sites and countries, the timing of the first sampling was determined by the flowering peak of dandelions (*Taraxacum officinale agg*. [L.] Weber ex F.H. Wigg). Each transect observation lasted 30 min. Thirty seconds was added for each collected specimen to account for handling time and to avoid undersampling sites with many bee individuals. To sample all target species when they were active, sampling only took place on days with temperatures >15°C, local wind speed < 5 m/s, little to no cloud cover, and no rain. All flower‐visiting bees observed in flowers were collected with a net or directly into a plastic vial and stored in 96% EtOH prior to identification. Bumble bee species in the *Bombus sensu stricto* subgenus (in this context *B. lucorum*, *B. terrestris*, *B. cryptarum*, and *B. magnus*) are cryptic and cannot be reliably identified morphologically (Carolan et al., 2012). Therefore, specimens in the *Bombus sensu stricto* subgenus were treated as 1 morphospecies, and all other bumble bees were identified to species. We pooled these samples to get estimates of bee species richness at each study site. We excluded records of kleptoparasites because their distribution is related to that of their hosts and, therefore, indirectly to environmental conditions. The data from Sydenham et al. (2024) consisted of 953 occurrences of 79 species of nonparasitic wild bees distributed across 68 wild bee communities in 2 regions, 1 in Denmark and 1 in Norway (Figure 1). The sampled bees belonged to 21 genera in 6 families: Melittidae, Andrenidae, Colletidae, Halictidae, Megachilidae, and Apidae. We used data from vegetation surveys conducted at each site by Sydenham et al. (2024). At each site, a vegetation survey was conducted in July, during which the occurrence of herbaceous plants in 10 1‐m^2^ vegetation survey plots placed regularly along the transect was recorded. As an indicator of local plant species richness, we counted the number of plant species in a site for which at least 1 interaction with wild bees had been recorded across the 68 sites. All fieldwork was conducted following the regulations of Norway and Denmark.

### Mapping LLS

We derived 2 maps of seminatural LLS showing all seminatural LLS (seminatural LLS hereafter) and forest edges only. We used the CLC+ back bone land cover map from the European Environment Agency (European Union, Copernicus Land Monitoring Service, 2022). The CLC+ is a 10‐m resolution land cover map with pixels classified according to 11 land‐use classes: sealed (class 1), woody, needle leaved trees (class 2); woody, broadleaved deciduous trees (class 3); woody, broadleaved evergreen trees (class 4); low‐growing woody plants, bushes, shrubs (class 5); permanent herbaceous (class 6); periodically herbaceous (largely equivalent to cropland) (class 7); lichens and mosses (class 8); non‐ and sparsely vegetated (class 9); water (class 10); and snow and ice (class 11). We identified seminatural LLS by using the as.lines, as.polygons, and rasterize functions in the terra package in R (Hijmans, 2023) to first polygonize all pixels classified as forest (CLC+ classes 2 and 3), low‐growing woody plants (class 5), grassland (class 6), and non‐ and sparsely vegetated (class 9) and then to convert these polygons into lines representing seminatural LLS. We followed the same procedure to produce a vector map that included only lines around forest edges (CLC+ classes 2 and 3). To estimate the length of nonforest seminatural LLS around study sites, we subtracted the length of forest edges from the length of all seminatural LLS.

### Provision of habitat by seminatural LLS

To determine whether seminatural LLS provided habitats for wild bees, we used Poisson generalized linear models in R (R Core Team, 2023) to assess whether wild bee species richness increased as the length of each of the 3 groups of LLS increased: all seminatural LLS, forest edges, or nonforest seminatural LLS in the surrounding landscape. We calculated the length of each type of LLS within radii of 250, 500, 750, 1000, 1250, and 1500 m around each sampling location. The size of the radius was chosen based on typical foraging ranges of bees (see Steffan‐Dewenter et al., 2002). To identify the spatial scale at which LLS had the strongest effect on bee species richness, we compared models in which the log‐transformed length of LLS was calculated within each radius and selected the model with the lowest Akaike information criterion, with a correction for small sample sizes (AICc value). The models were formulated using the following R syntax:

(1) $$\begin{array}{ccc} & & Y∼\log\left(\mathrm{LL}S_{\mathrm{radius}}\right)+\mathrm{plant}\;\mathrm{richness}+\log\left(\mathrm{grasslan}d_{\mathrm{radius}}\right)\\ & & +\mathrm{country}+\mathrm{Xcrds}+\mathrm{Ycrds}, \end{array}$$

where *Y* is species richness of bees sampled in a site; log(LLS_radius_) is the log‐transformed length of all seminatural LLS, forest edges only, or nonforest seminatural LLS in the circular radius. Plant richness was the site‐specific flowering plant species richness, included to control for site‐specific differences in habitat quality. Log(grassland_radius_) was the log‐transformed proportion of grassland (CLC+ permanent herbaceous) within the radius and was included to control for potential confounding effects of habitat availability in the surrounding landscape. Country is a categorical variable used to define the country of the samples (Denmark vs. Norway) and was included to control for potential country‐specific differences in bee species richness. The *X*crds and *Y*crds are the longitudinal and latitudinal coordinates, respectively, of each site and were used to correct for potential residual spatial autocorrelation. We used AICc to identify the best performing models (lowest AICc) and Moran's *I* autocorrelation index in the R package ape (Paradis & Schliep, 2019) to determine whether Pearson residuals were spatially correlated. We used DHARMa residuals (Hartig, 2022) to test for overdispersion and to assess residual distributions from the models with the lowest AICc.

For the selected models, we used likelihood ratio test (LRT) statistics to assess the statistical significance of LLS variables and included an interaction term between country and the log‐transformed length of the LLS to test for potential country‐specific differences in the response of bee species richness to the length of the LLS.

### Seminatural LLS and dispersal corridors

To determine whether seminatural LLS provided dispersal corridors for wild bees, we determined whether the bee species compositional similarity between site pairs was better explained by between‐site distances along hypothesized dispersal routes (i.e., LCPs) that tracked seminatural LLS, or forest edges, than by geographic distances. Because we were interested in how land‐use conditions affect habitat connectivity, we restricted our analyses to site pair comparisons in the same country.

We calculated species compositional similarity between sites as 1−BC, where BC (Bray−Curtis dissimilarity) was computed using the vegdist function in Vegan (Oksanen et al., 2022). Similarity was calculated for all wild bees, for solitary bees only, and for plant species. The BC dissimilarity was based on binary presence‐absence matrices, calculated as (*A*+*B*−2*J*)/(*A*+*B*), where *A* and *B* are species richness at sites *i* and *j*, and *J* is the total number of species. Prior to calculating BC dissimilarity for solitary bees, we added a dummy species to all sites to reduce zero inflation (Graham et al., 2021). We assembled a data frame with columns for site *i* and *j* identities, wild bee, solitary bee, and plant species compositional similarities, plant richness at each site, and the country of each site pair.

For each site pair (*i*, *j*), we calculated geographic distance with the distance function in terra (Hijmans, 2023) in R. We used the maps of seminatural LLS and forest edges to identify the shortest dispersal corridors that tracked these features between sites. We used rasterize in terra (Hijmans, 2023) to rasterize the spatial lines representing seminatural LLS or forest edges into the 10‐m projection of the CLC+ maps. Estimating LCPs requires an adjacency matrix defined from all cells in a raster. For computational efficiency, we aggregated the 10‐m resolution LLS and forest edge maps into 100‐m resolutions; cell values represented the proportion of 10‐m cells classified as a seminatural LLS or forest edge. We estimated LCPs between site pairs with the create_cs function (neighbors = 8) from the leastcostpath package (Lewis, 2023). Conductance surfaces were derived from 100‐m rasters of seminatural LLS and forest edges; an 8‐cell neighborhood defined adjacent cells (N, NE, E, SE, S, SW, W, NW). Higher proportions of these features were interpreted as increasing landscape permeability for movement.

We adapted the function create_lcp in leastcostpath (Lewis, 2023) to identify the LCPs between site pairs based on cost surfaces. We used the igraph package in R (Csardi & Nepusz 2006; Csárdi et al., 2024) to identify LCPs. We used the graph_from_adjacency_matrix function with mode = min and weighted = true to create a weighted graph from the conductance matrix between cells in the cost surface. As in the create_lcp function (Lewis, 2023), we transformed the weights (1/*x*) to convert conductance values into costs. Costs increased exponentially as the proportion of LLS decreased. For each site pair in the same country, the shortest_paths function in igraph identified raster cells along the shortest path, which was converted into a spatial lines object (spatvector). Lengths of the LCPs were calculated using the perim function from terra (Hijmans, 2023). The LCP lengths alone may not fully quantify the cost of movement across the landscape (Etherington & Holland, 2013). To estimate movement costs, the 100‐m habitat edge rasters were transformed (1−*x*), and pixel values along each path were summed.

Visual inspection showed a unimodal (hump‐shaped) response (Appendix S1) of wild bee species compositional similarity to geographic distance (1–105 km, mean [SD] = 41.8 km [24.4]). This pattern likely arises because species similarity initially declines as dispersal distance increases due to dispersal limitations and changes in land‐use, and then increases at larger distances where spatially clustered land‐use types support similar species pools (Newbold et al., 2016). To focus on data for which similarity decreased as distance increased, we performed a sensitivity analysis to identify the maximum distance beyond which similarity increased. We created 20 data subsets, limiting maximum distances to 5 km increments (10–105 km), and used Gaussian generalized linear mixed models (GLMMs) from the glmmTMB R package (Brooks et al., 2017) to model compositional similarity as a function of untransformed distance or a second‐order polynomial transformation with the *i*th and *j*th site identities as random intercept terms. We compared models by using ΔAICc; negative values favored the untransformed model. The analysis was performed separately for each country and across pooled data. For each country, we included pairwise distances below the threshold where the untransformed model performed best (ΔAICc < 0) or equally well as the transformed model (|ΔAICc| < 2) to retain as much data as possible for further analyses (Appendix S1).

For the Danish data, we included all pairwise distances from 0 to 79 km, whereas for the Norwegian data, analyses were restricted to site pairs no farther apart 55 km (Appendix S1). We used DHARMa residual plots to compare the goodness of fit of Gaussian GLMMs and t_family GLMMs to test the relationships between wild bee and solitary bee species compositional similarity and geographic distance or LCP lengths and costs. We proceeded with the t_family GLMMs because they performed best for both all wild bees and solitary bees. Analyses were run separately for each country to account for potential country‐specific differences in distance decay relationships. The models were formulated using the following R syntax:

(2) $$\begin{array}{ccc} & & Y∼\mathrm{distanc}e_{ij}+\mathrm{plantS}R_{i}\times \mathrm{plantS}R_{j}+\mathrm{plant}\;\mathrm{com}sim_{ij}\\ & & +\left(1|\mathrm{site}\;i\right)+\left(1|\mathrm{site}\;j\right), \end{array}$$

where *Y* is either the wild bee species or solitary bee species compositional similarity between the *i*th and *j*th site; distance*_ij_* is either the geographic distance, LCP lengths following all seminatural LLS, forest edges, or travel‐costs along the 2 LCPs; plantSR is the plant species richness of the *i*th or *j*th site; and plant com sim is the plant species compositional similarity between sites *i* and *j*, which were included to control for site‐specific differences in plant composition and its potentially confounding effect on bee species compositional similarity. The random intercepts (1|site *i*) and (1|site *j*) were included to correct for site‐specific differences in the average similarity to other sites. We used DHARMa (Hartig, 2022) residual plots to confirm that residual distributions aligned with model assumptions. We used AICc to compare all models from the same country against a null model that did not include the distance*_ij_* parameter.

## RESULTS

Across all sites, bee species richness ranged from 2 to 18 (mean [SD] = 9.34 [4.05]). For Norwegian sites, the range was 2−18 (mean = 10.37 [4.41]), and for Danish sites, 2−14 (mean = 7.78 [2.83]). Despite that sampling occurred along roadsides, the data contained several species that are typically associated with extensively managed grasslands, such as *Andrena hattorfiana* and *A. humilis*. A full list of the bee species sampled and the number of sites they occurred in is in Appendix S1.

### Seminatural LLS provision of habitat

**FIGURE 2:**
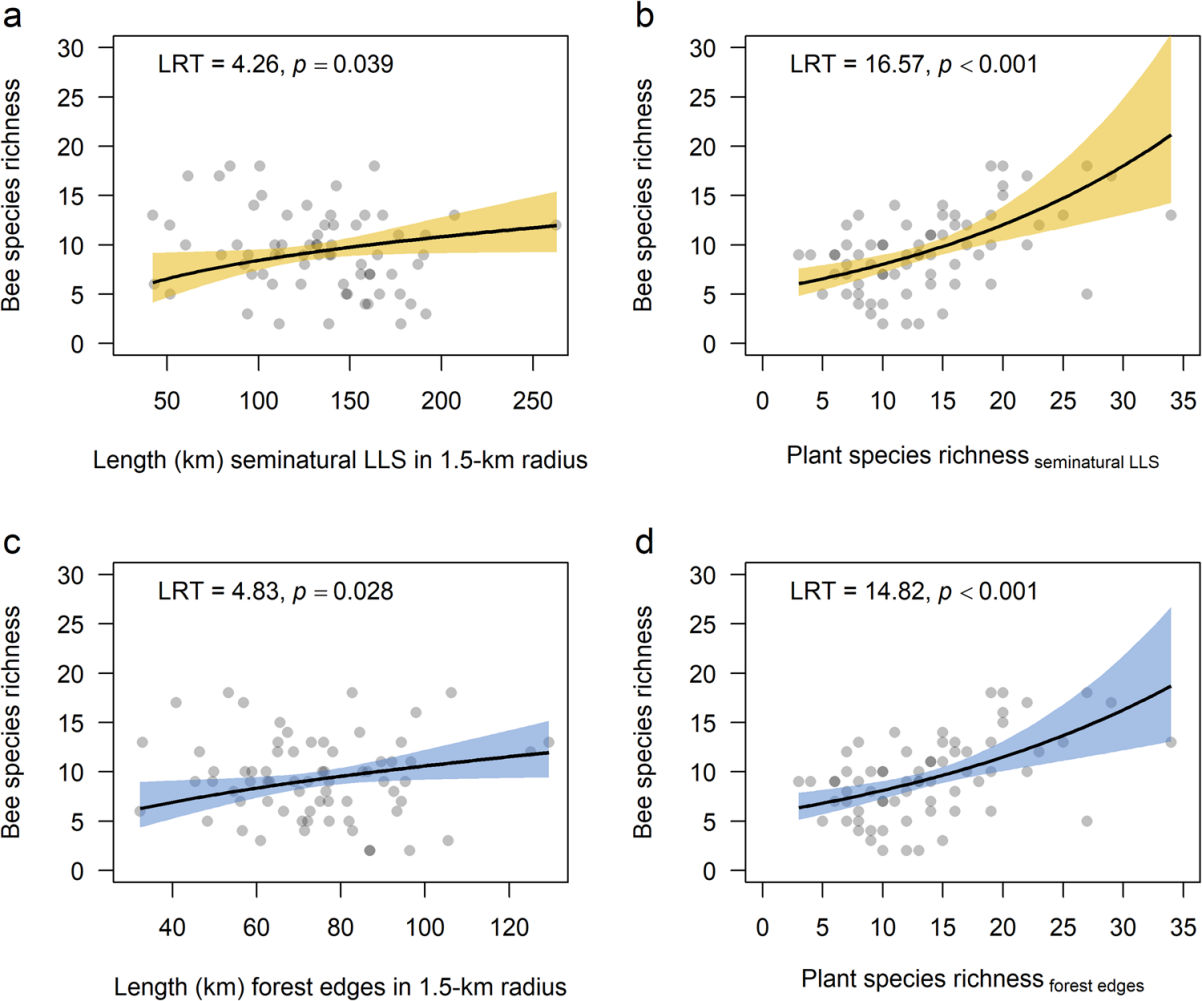
Wild bee species richness relative to (a) length of seminatural habitat edges, (b) plant species richness within 1.5 km of sampling sites, (c) length of forest edges, and (d) local plant species richness (lines, regression lines from Poisson generalized linear models; shading, 95% confidence intervals). Likelihood ratio test (LRT) values and *p* values are from drop1 tests. Full model details are in Appendix S1.

Wild bee species richness increased as the length of seminatural LLS in the surrounding landscape increased, suggesting that seminatural LLS provided habitat resources for wild bees (Appendix S1). The relationship between bee species richness and the length of seminatural LLS was the strongest when LLS lengths were calculated within the 1500‐m radius (AICc = 373.4) (Figure 2a & Table 1). The controlling variable, plant species richness in sites, was a strong predictor of bee species richness (Figure 2b). For the length of forest edges, the relationship was the strongest when measured within the 1000‐m radius (Figure 2c & Table 1) (AICc = 372.5), but for comparability with the seminatural LLS model, we focused on the 1500‐m radius (AICc = 372.8) (Table 1).

**TABLE 1:** Comparisons of Poisson generalized linear models (GLMs) of wild bee species richness as a function of seminatural linear landscape structure (LLS) length in circular areas around sampling sites.^*^

Sampling area radius (m)	AICc _all LLS_	AICc _forest LLS_	AICc _nonforest LLS_
250	374.7	378.8	373.2
: 500	378.1	379.0	378.3
: 750	376.7	375.2	377.2
: 1000	373.9	372.5	375.3
: 1250	373.8	373.4	374.3
: 1500	373.4	372.8	374.2

* The plant species richness in sites, proportion of grassland in sampling area, and site‐specific spatial coordinates (EPSG:3035) were included in the models to correct for potential confounding effects from environmental conditions and spatial autocorrelation. Comparisons were made using Akaike information criterion (AICc) values across models in which lengths of linear landscape structures were identified using all seminatural LLSs (AICc _All LLS_), forest edges only (AICc _forest LLS_), or all nonforest edge habitats (AICc _nonforest LLS_). Poisson GLMs of wild bee species richness were compared with null models that did not include any LLSs (AICc _null model_).

Also, in these models, the controlling variable, plant species richness, was a strong predictor of bee species richness (Figure 2d). When forest edges were excluded from the seminatural LLS, the model performed slightly worse, but not significantly so (AICc = 374.2) (Table 1). Moran's *I* autocorrelation test showed no signs of spatial dependency of residuals for any of the 3 models: all seminatural LLS (*p* = 0.817) and forest edges only (*p =* 0.484); nonforest seminatural LLS (*p =* 0.840). The relationship between bee species richness and the length of seminatural LLS (interaction term country × log[length of seminatural LLS_1500m radius_], df = 1, LRT = 2.18, *p =* 0.14), forest edges (interaction term country × log[length of forest edges_1500‐m radius_], df = 1, LRT = 1.14, *p =* 0.28), or nonforest seminatural LLS (interaction term country × log[length of nonforest seminatural LLS_1500‐m radius_], df = 1, LRT = 1.82, *p =* 0.17) did not differ significantly between the 2 countries. We, therefore, did not include these interaction terms in the final models.

LRTs of the main effects in the 1500‐m models showed that the contributions of the length of seminatural LLS (df = 1, LRT = 4.26, *p =* 0.04) and forest edges (df = 1, LRT = 4.83, *p =* 0.03) to explaining differences in bee species richness across sites were statistically significant, whereas length of nonforest seminatural LLS was not (df = 1, LRT = 3.47, *p =* 0.06). Bee species richness increased slightly more as the length of forest edges increased (β_forest edge length_ = 0.47, log‐link scale) compared with the length of all seminatural LLS (β_seminatural LLS length_ = 0.36, log‐link scale) and nonforest seminatural LLS (β_nonforest seminatural LLS length_ = 0.13, log‐link scale), such that doubling the length of forest edges would result in a 38% increase of bee species richness compared with a 28% or 10% increase when doubling the length of all seminatural LLS or nonforest seminatural LLS, respectively.

### Seminatural LLS and dispersal corridors

Bee species compositional similarity (i.e., proportion of bee species co‐occurring in 2 sites) ranged from 0 to 0.89 (mean [SD] = 0.35 [0.15]) in Denmark and from 0 to 0.80 (mean = 0.36 [0.15]) in Norway (including on‐site pairs < 55 km apart). The LCP corridors defined by seminatural LLS were 1.07−2.11 times longer (mean = 1.38 [0.14]) than geographic distances for the Danish site pairs and 1.02−3.67 times longer (mean = 1.46 [0.24]) for the Norwegian site pairs. When LCPs were defined by forest edges, they were 1.07−2.57 times longer (mean = 1.55 [0.25]) than geographic distances in Denmark, and 1.08−3.67 times longer (mean = 1.51 [0.24]) in Norway. LCP lengths for all seminatural LLS ranged from 0 to 110 km in Denmark (mean = 48.46 [30.09]) and from 1 to 109 km in Norway (mean = 43.03 [27.93]). For forest edges, LCP lengths ranged from 0 to 119 km in Denmark (mean = 54.40 [33.66]) and from 1 to 108 km in Norway (mean = 43.53 [27.43]).

**FIGURE 3:**
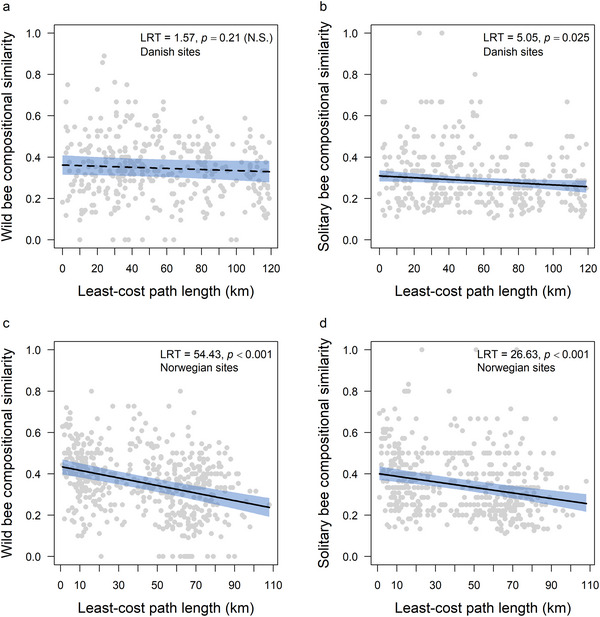
(a, c) Wild and (b, d) solitary bee species compositional similarity as least‐cost path length along forest edges increases in Denmark and Norway sampling sites (lines, regression lines from generalized linear models; shading, 95% confidence intervals; N.S., not significant; controlling variables held at mean values). Likelihood ratio test (LRT) and *p* values are from drop1 tests.

When considering all wild bee species, we found no evidence that bee species compositional similarity was geographically structured across the Danish sites (Appendix S1). The null model, which did not include any spatial distance measure, provided an equally good fit to the data (AICc = −432.6) as the models that included the geographic distance (AICc = −431.4), length of LCP defined by seminatural LLS (AICc = −432.2), length of LCP defined by forest edges (AICc = −432.0) (Figure 3a), cost of LCP defined by seminatural LLS (AICc = −432.2), and cost of LCP defined by forest edges (AICc = −432.1). However, when considering only solitary bees (Appendix S1), the null model provided the poorest fit to the data (AICc = −423.2), followed by the geographic distance model (AICc = −423.8), which was outperformed by models that included length of LCP defined by seminatural LLS (AICc = −425.4, ΔAICc = 1.6), length of LCP defined by forest edges (Figure 3b, AICc = −426.2, ΔAICc = 2.4), cost of LCP defined by seminatural LLS (AICc = −425.5, ΔAICc = 1.7), and cost of LCP defined by forest edges (AICc = −426.3, ΔAICc = 2.5). LRTs of the forest edge LCP length model showed that solitary bee species compositional similarity decreased as LCP length increased (*z* = −2.25, df = 1, LRT = 5.05, *p =* 0.02), increased as plant species compositional similarity increased (*z* = 3.14, df = 1, LRT = 9.53, *p<* 0.01), and was not related to the interaction between plant species richness in site pairs (*z* = 0.24, df = 1, LRT = 0.06, *p =* 0.81).

For the Norwegian sites, wild bee species compositional similarity was geographically structured regardless of whether bumblebees were included in the analyses (Appendix S1). When including bumblebees, the null model yielded the poorest fit to the data (AICc = −557.4), followed by the geographic distance model (AICc = −604.5) and the cost of LCP defined by the seminatural LLS (AICc = −601.5). The best performing models, which all outperformed the geographic distance model, were the length of LCP defined by seminatural LLS (AICc = −607.7, ΔAICc = 3.2), length of LCP defined by forest edges (AICc = −609.7, ΔAICc = 5.2) (Figure 3c), and cost of LCP defined by forest edges (AICc = −608.9, ΔAICc = 4.4). When restricting the analyses to solitary bees, the null model also provided the poorest fit to the data (AICc = −492.0), followed by the geographic distance model (AICc = −512.9). The geographic distance model was outperformed by models that included length of LCP defined by seminatural LLS (AICc = −516.3, ΔAICc = 3.4), length of LCP defined by forest edges (AICc = −516.6, ΔAICc = 3.7) (Figure 3d), cost of LCP defined by seminatural LLS (AICc = −517.9, ΔAICc = 5.0), and cost of LCP defined by forest edges, which provided the best fit (AICc = −519.6, ΔAICc = 6.7). LRTs of the forest edge LCP length models showed that wild bee species compositional similarity decreased as LCP length increased (*z =* −7.60, df = 1, LRT = 54.43, *p<* 0.01), increased as plant species compositional similarity increased (*z* = 2.28, df = 1, LRT = 5.22, *p =* 0.02), and the interaction between plant species richness in site pairs increased (*z* = 2.02, df = 1, LRT = 4.04, *p =* 0.04). Similarly, when considering only solitary bees in Norway, species compositional similarity decreased as LCP length increased (*z =* −5.22, df = 1, LRT = 26.63, *p<* 0.01) and increased as the interaction between plant species richness in the 2 sites (*z =* 3.39, df = 1, LRT = 11.21, *p<* 0.01) increased but did not depend on plant species compositional similarity (*z =* −0.29, df = 1, LRT = 0.08, *p =* 0.77).

## DISCUSSION

We found that the presence of seminatural LLS not only increased bee species richness but also the connectivity between bee communities. Indeed, the data used in this study were collected along roadsides but still contained several bee species associated with seminatural grasslands (Appendix S1), suggesting that the roadside habitats sampled provided important resources for the local bee communities. Our findings highlight the dual role of seminatural LLS, particularly forest edges, as both vital habitats and dispersal conduits for wild bees in intensively managed temperate landscapes.

Our findings align with previous research showing that compositional similarity in pollinator communities decreases as geographic distance increases, suggesting dispersal limitations. This pattern, known as distance decay, has been observed in plant‐pollinator networks, where pollinator species turnover increases as distance increases (Carstensen et al., 2014), and in bee surveys, where the likelihood of a bee species occurring in a habitat declines as the distance from its known occurrence increases (Sydenham et al., 2017). In grassland habitats, indices of pollinator species diversity, such as species evenness, are highest in habitats surrounded by large patches of grassland to which geographic distances are short, indicating a high degree of habitat connectivity (Marini et al., 2014). Our findings that LCP lengths provided better predictors of bee species compositional similarity than geographic distance align with the review of Vasiliev and Greenwood (2023) in suggesting that incorporating ecological and landscape factors in distance measures offers a more comprehensive understanding of connectivity in pollinator communities. Additionally, including landscape factors, such as seminatural LLS, in estimates of habitat connectivity makes it possible to identify potentially important dispersal corridors through intensively managed landscapes.

Habitat resources are often a limiting factor for bee species diversity in intensively managed agricultural (Le Féon et al., 2010; Steffan‐Dewenter et al., 2002) and forested landscapes (Winfree et al., 2007). In this particular landscape context, seminatural LLS may provide nesting and foraging resources and consequently increase dispersal rates along dispersal routes (reviewed in Vasiliev & Greenwood, 2023). Seminatural LLS can occur, for example, in the form of sun‐exposed forest edges (Proesmans et al., 2019; Sydenham et al., 2014), road verges (Eckerter et al., 2023; Hopwood, 2008; Johansen et al., 2022), grassland edges (Cole et al., 2015), edges around sparsely vegetated areas, such as quarries (Heneberg & Bogusch, 2020), and power line clearings (Eldegard et al., 2015; Wagner et al., 2019). Our findings suggest that forest edges are particularly important in contributing to habitat resources for bees. That bee diversity is often lower in forested microhabitat sites compared with clearcuts or forest edges (Mullally et al., 2019; Nielsen & Totland, 2014) suggests that the forest edge—and not the forest itself—provides resources for bees. Indeed, species richness and abundance of flower visitors decreased as distance to forest edges increased (Bailey et al., 2014; Ricketts, 2004). Other transient habitat patches, such as clearcuts, in forested landscapes can provide important floral resources for pollinators (Nielsen & Totland, 2014; Rubene et al., 2015), but their value as pollinator habitat quickly diminishes due to regrowth and canopy closure (Zitomer et al., 2023). By contrast, our findings suggest that seminatural LLS can provide persistent habitat resources for wild bees in intensively managed landscapes.

However, wild bees require large quantities of pollen to sustain their populations, with many bee species requiring the pollen from more than 30 flowers to rear a single larva (Müller et al., 2006). To support wild bee populations, a landscape must also provide enough nectar to meet foragers’ energy needs and help overwintering bumble bee queens build carbohydrate stores (reviewed in Woodard & Jha, 2017). Compared with core habitats, such as seminatural grasslands, the limited size of seminatural LLS restricts the resources they can provide and, consequently, the bee species richness they can sustain for extended periods of time (Johansen et al., 2022). We found that bee species richness in sites increased as local plant species richness increased, and that bee communities from plant‐species‐rich sites and sites with similar plant species compositions often had more similar bee communities than sites that differed in plant species richness or composition. The importance of plant species richness as an indicator of habitat quality of LLS has also been found for bumblebee and butterfly species richness along Swedish roadsides (Horstmann et al., 2023). However, due to the high pollen requirements of many bee species and their restricted foraging range (Zurbuchen et al., 2010), seminatural LLS do not reduce the need to conserve and restore larger seminatural grassland habitats (Von Königslöw et al., 2021).

Although seminatural LLS may not be able to replace seminatural grasslands (Johansen et al., 2022; Von Königslöw et al., 2021), our findings align with those of Menz et al. (2011) in suggesting that they may function as important steppingstones and increase habitat connectivity. Management schemes for improving habitat conditions for wild bees along seminatural LLS can increase their function as dispersal corridors. For mown areas, selective mowing can be adopted to prevent encroachment and leave parts of the habitat as refuges for pollinators (Buri et al., 2014). Timing mowing to the flowering time of the local flora can ensure floral resources are present when bees require them (Johansen et al., 2019). In cases where plant diversity is initially low, seeding the seminatural LLS with native plant species (Hopwood, 2008; Mitchell et al., 2024), ideally of regional provenance to preserve local genetic diversity (Kaulfuß & Reisch, 2019), may be required for the area to provide resources for bees. Allowing the formation of grass tussocks can improve nesting conditions for bumblebees because several species nest under thick grass swards (O'Connor et al., 2017). In addition to improving plant species richness and bumblebee nesting conditions through altered mowing regimes, nesting sites for ground nesting bees can be introduced in the form of sand pockets (Fortel et al., 2016). Along grassland and forest edges, it is possible to cater to cavity nesting bees by allowing dead wood to gather (Westerfelt et al., 2015) and by preserving or building stone drywalls (Xie et al., 2020). Implementing these management actions will incur a cost for managers and should be directed to where they will have the desired effect. Our findings suggest that maps of LCPs between pollinator habitats of conservation priority can be used to identify potentially important dispersal corridors along which management actions should be prioritized.

Bee species are likely to differ in their dispersal abilities. There is, for instance, some evidence that the dispersal ability of bee species increases as bee body size increases (López‐Uribe et al., 2019) and that the response of bees to habitat loss depends on bee dispersal abilities (Bommarco et al., 2010). Indeed, it was only when focusing analyses on solitary bees, which we expect to be poorer dispersers than bumblebees, that we found a significant distance decay in bee species compositional similarity in Denmark. Our findings suggest that small‐bodied bees not only have shorter foraging ranges than larger species (Greenleaf et al., 2007; Zurbuchen et al., 2010), but may also face greater dispersal limitations, making them more vulnerable to habitat isolation.

We focused on seminatural LLS and their role as potential dispersal corridors, and by doing so, treated other land cover types in the landscape as having a high resistance to movement. As shown by Beduschi et al. (2018), the resistance to movement is likely to differ between forests, fields, and extensively managed grasslands, with extensively managed grasslands particularly facilitating dispersal. Because we did not include extensively managed grasslands in our estimates of LCP lengths, it is possible that we overestimated the dispersal distance between habitats. Still, when comparing our models of bee species compositional similarity, models where grassland edges were included when estimating LCPs did not outperform the forest edge‐based LCPs, suggesting that including grasslands as potential corridors would not significantly improve the models for connectivity. The relatively coarse thematic resolution of the grassland layer used in our models, where all permanent herbaceous areas are classified as grassland, may also have led to the underestimation of the importance of extensively managed grasslands (Marshall et al., 2021). In regions where habitat type maps with a higher thematic resolution are available, including species‐rich habitat types, such as extensively managed grasslands (Von Königslöw et al., 2021), as potential dispersal corridors, may further improve the accuracy of models for identifying potential dispersal corridors.

We used the bee species compositional similarity between sites as measures of connectivity. The compositional similarity between sites depends on the sample size in site pairs, and undersampling may inflate estimates of similarity and dissimilarity (Beck et al., 2013; Stier et al., 2016). Despite limiting our sampling effort to 3 surveys per site and the strong influence of local plant species richness on bee species richness and compositional similarity, we still detected a statistically significant relationship between LCP lengths and habitat connectivity estimated from compositional similarity. This held true even after accounting for differences in plant species richness and composition between sites. Moreover, for solitary bees, which can be expected to be more dispersal‐limited than bumblebees because of their smaller size (López‐Uribe et al., 2019), we found a decrease in compositional similarity with LCP lengths in both Danish and Norwegian study systems. We, therefore, find it unlikely that the decrease in species compositional similarity with LCP length, which was stronger than with geographic distance, would reflect a statistical bias.

Our results suggest that seminatural LLS, and particularly forest edges, contribute to enhancing connectivity among bee communities in intensively managed temperate landscapes. However, the value of such edges as habitat depends on their plant species richness. While implementing management actions, such as altered mowing regimes, to increase plant species richness in seminatural LLS is costly, LCP analyses can provide an efficient tool for identifying important dispersal corridors along which management actions should be prioritized. Considering dispersal corridors as an integrated part of pollinator conservation planning will allow the establishment of networks of high‐quality habitats where the longevity of populations of pollinators can be maintained through dispersal dynamics.

## AUTHOR CONTRIBUTIONS

Conceptualization: M.A.K.S. (lead), A.N., and B.S.; data curation: M.A.K.S., Y.L.D. (coleads), H.B.M., and M.S.T; formal analysis: M.A.K.S. (lead); funding acquisition: M.A.K.S. (lead), A.N., Y.L.D., and C.R.; investigation: M.A.K.S., Y.L.D. (coleads), H.B.M., and M.S.T.; methodology: M.A.K.S. (lead), Y.L.D., and C.R.; project administration: M.A.K.S.; validation: M.A.K.S.; visualization: M.A.K.S.; writing: original draft M.A.K.S. (lead), review and editing M.A.K.S. (lead), A.N., Y.L.D., H.B.M., C.R., M.S.T., and B.S.

## Supporting information



Supplementary Material

## Data Availability

Data and R code associated with this paper are deposited on Zenodo (10.5281/zenodo.15277805).
