# Supplementary material for: Role of forest edges and other seminatural linear landscape features in structuring wild bee habitat connectivity in intensively managed landscapes
Source: Conserv Biol. 2025 Sep 30;39(6):e70152. doi: 10.1111/cobi.70152 (PMC12658934; doi:10.1111/cobi.70152)
Supplement: Supplementary file 1 — Supplementary Material [file COBI-39-e70152-s001.docx]

**Appendix S1: Tables S1*– S6 and Figure S1***

**Table S1** Overview of the bee species sampled and the number of Danish and Norwegian study sites each species occurred in.

| Bee species | Danish Sites | Norwegian Sites |
| --- | --- | --- |
| *Andrena bicolor* | 1 | 1 |
| *Andrena chrysosceles* | 6 | 0 |
| *Andrena cineraria* | 3 | 0 |
| *Andrena denticulata* | 2 | 12 |
| *Andrena fucata* | 0 | 2 |
| *Andrena fulva* | 1 | 0 |
| *Andrena fulvida* | 0 | 1 |
| *Andrena haemorrhoa* | 9 | 4 |
| *Andrena hattorfiana* | 4 | 0 |
| *Andrena helvola* | 2 | 0 |
| *Andrena humilis* | 1 | 0 |
| *Andrena labiata* | 1 | 0 |
| *Andrena lathyri* | 0 | 4 |
| *Andrena nigriceps* | 0 | 2 |
| *Andrena nigroaenea* | 6 | 1 |
| *Andrena semilaevis* | 5 | 0 |
| *Andrena subopaca* | 5 | 13 |
| *Andrena tarsata* | 0 | 1 |
| *Andrena wilkella* | 5 | 3 |
| *Anthidium manicatum* | 1 | 0 |
| *Anthidium punctatum* | 0 | 2 |
| *Bombus distinguendus* | 0 | 3 |
| *Bombus hortorum* | 9 | 21 |
| *Bombus humilis* | 0 | 14 |
| *Bombus hypnorum* | 5 | 10 |
| *Bombus lapidarius* | 20 | 18 |
| *Bombus pascuorum* | 24 | 34 |
| *Bombus pratorum* | 8 | 22 |
| *Bombus ruderarius* | 0 | 7 |
| *Bombus sensu strictu* | 20 | 33 |
| *Bombus soroeensis* | 1 | 18 |
| *Bombus subterraneus* | 0 | 4 |
| *Bombus sylvarum* | 0 | 19 |
| *Bombus wurflenii* | 0 | 4 |
| *Ceratina cyanea* | 0 | 1 |
| *Chelostoma florisomne* | 0 | 3 |
| *Colletes cunicularius* | 1 | 0 |
| *Colletes daviesanus* | 2 | 12 |
| *Colletes fodiens* | 3 | 0 |
| *Colletes similis* | 1 | 3 |
| *Dasypoda hirtipes* | 6 | 0 |
| *Dufourea dentiventris* | 0 | 2 |
| *Eucera longicornis* | 0 | 12 |
| *Halictus rubicundus* | 1 | 21 |
| *Halictus tumulorum* | 6 | 19 |
| *Heriades truncorum* | 0 | 1 |
| *Hoplitis claviventris* | 0 | 3 |
| *Hylaeus brevicornis* | 1 | 2 |
| *Hylaeus communis* | 0 | 5 |
| *Hylaeus confusus* | 2 | 8 |
| *Hylaeus dilatatus* | 1 | 0 |
| *Hylaeus hyalinatus* | 0 | 2 |
| *Hylaeus pectoralis* | 1 | 0 |
| *Hylaeus rinki* | 0 | 4 |
| *Lasioglossum albipes* | 3 | 10 |
| *Lasioglossum calceatum* | 4 | 2 |
| *Lasioglossum fratellum* | 0 | 8 |
| *Lasioglossum fulvicorne* | 0 | 1 |
| *Lasioglossum lativentre* | 3 | 0 |
| *Lasioglossum leucopus* | 5 | 12 |
| *Lasioglossum leucozonium* | 6 | 0 |
| *Lasioglossum morio* | 1 | 0 |
| *Lasioglossum punctatissimum* | 1 | 0 |
| *Lasioglossum quadrinotatum* | 8 | 0 |
| *Lasioglossum rufitarse* | 0 | 6 |
| *Lasioglossum sexstrigatum* | 1 | 0 |
| *Lasioglossum villosulum* | 5 | 1 |
| *Lasioglossum zonulum* | 0 | 7 |
| *Macropis europaea* | 0 | 3 |
| *Megachile alpicola* | 0 | 5 |
| *Megachile circumcincta* | 0 | 3 |
| *Megachile nigriventris* | 0 | 1 |
| *Megachile versicolor* | 0 | 1 |
| *Megachile willughbiella* | 3 | 8 |
| *Melitta haemorrhoidalis* | 3 | 0 |
| *Osmia leaiana* | 2 | 0 |
| *Osmia uncinata* | 0 | 1 |
| *Panurgus calcaratus* | 1 | 4 |
| *Trachusa byssina* | 0 | 1 |

**Table S2.** Poisson GLM outputs testing wild bee species richness against the lengths of all seminatural LLS, forest edges, and non-forest seminatural LLS within buffers 1500m around study sites. The table includes likelihood ratio test (LRT) results comparing models with and without linear landscape structures, estimated regression slopes (Est.), and effect sizes (z-values). Models controlled for plant species richness, country, longitude, latitude, and grassland proportion within the buffer. Intercept statistics are omitted.

| Wild bee species richness | | |  |  |  |  |  |
| --- | --- | --- | --- | --- | --- | --- | --- |
|  | Seminatural LLS | | Est. | z-value | AIC | LRT | p |
|  |  | No variables removed (null) |  |  | 371.53 |  |  |
|  |  | Intercept | - 23.75 | - 2.13 |  |  |  |
|  |  | Plant species richness | 0.04 | 4.10 | 385.69 | 16.57 | < 0.01 |
|  |  | Country (Norway vs. Denmark) | - 0.62 | -0.64 | 369.82 | 0.41 | 0.52 |
|  |  | Longitude | < 0.01 | 1.80 | 372.72 | 3.24 | 0.07 |
|  |  | Latitude | < 0.01 | 0.43 | 369.61 | 0.18 | 0.67 |
|  |  | Log(Prop. Grassland 1500m) | 0.03 | 0.26 | 369.59 | 0.07 | 0.79 |
|  |  | Log(All LLS 1500m) | 0.36 | 2.06 | 374.47 | 4.26 | 0.04 |
|  |  |  |  |  |  |  |  |
|  | Forest edges | | Est. | z-value | AIC | LRT | p |
|  |  | No variables removed (null) |  |  | 372.31 |  |  |
|  |  | Intercept | -26.79 | -2.28 |  |  |  |
|  |  | Plant species richness | 0.03 | 3.89 | 383.77 | 14.82 | < 0.01 |
|  |  | Country (Norway vs. Denmark) | -1.06 | -1.04 | 370.04 | 1.08 | 0.30 |
|  |  | Longitude | < 0.01 | 1.71 | 371.86 | 2.90 | 0.09 |
|  |  | Latitude | < 0.01 | 0.89 | 369.74 | 0.78 | 0.38 |
|  |  | Log(Prop. Grassland 1500m) | -0.02 | -0.15 | 368.98 | 0.02 | 0.88 |
|  |  | Log(Forest edges 1500m) | 0.47 | 2.21 | 373.78 | 4.83 | 0.03 |
|  |  |  |  |  |  |  |  |
|  | Non-forest seminatural LLS | | Est. | z-value | AIC | LRT | p |
|  |  | No variables removed (null) |  |  | 372.31 |  |  |
|  |  | Intercept | -15.02 | -1.67 |  |  |  |
|  |  | Plant species richness | 0.04 | 4.04 | 385.88 | 15.86 | < 0.01 |
|  |  | Country (Norway vs. Denmark) | -0.36 | -0.38 | 370.54 | 0.14 | 0.70 |
|  |  | Longitude | < 0.01 | 1.48 | 372.54 | 2.19 | 0.14 |
|  |  | Latitude | < 0.01 | 0.25 | 370.46 | 0.06 | 0.81 |
|  |  | Log(Prop. Grassland 1500m) | 0.11 | 1.00 | 371.32 | 1.01 | 0.31 |
|  |  | Log(Non-Forest Edges 1500m) | 0.13 | 1.83 | 374.47 | 3.47 | 0.06 |

**Table S3.** T-family GLMM results testing wild bee species compositional similarity between Danish site pairs across various distance measures (geographic, least-cost paths via seminatural LLS or forest edges). A null model without distance was included for comparison. The table shows likelihood ratio test statistics (LRT, AIC, p), estimated slopes (Est.), and effect sizes (z-values). Models control for plant species composition and richness differences via an interaction term. Intercept statistics are omitted.

| Danish sites: All wild bees | | | | | | | | |
| --- | --- | --- | --- | --- | --- | --- | --- | --- |
|  |  | Fixed Effect - null model | | Est. | z-value | AIC | LRT | p |
|  |  |  | No variables removed (null) | | | -433.12 |  |  |
|  |  |  | Intercept | 0.32 | 12.30 |  |  |  |
|  |  |  | Plant comp. similarity | 0.09 | 1.74 | -432.1 | 3.01 | 0.083 |
|  |  |  | scale(Plant richness site i):scale(Plant richness site j) | <0.01 | -0.08 | -435.11 | 0.01 | 0.939 |
|  |  | Random effects (obs = 351) | | Name | Variance | Std. Dev. |  |  |
|  |  |  | Site i (n = 26) | Intercept | 0.004 | 0.062 |  |  |
|  |  |  | Site j (n = 26) | Intercept | 0.006 | 0.075 |  |  |
|  |  | Fixed Effect - Geographic distance model | | Est. | z-value | AIC | LRT | p |
|  |  |  | No variables removed (null) | | | -432.09 |  |  |
|  |  |  | Intercept | 0.32 | 12.42 |  |  |  |
|  |  |  | scale(Geographic distance) | -0.01 | -0.99 | -433.12 | 0.98 | 0.323 |
|  |  |  | Plant comp. similarity | 0.08 | 1.55 | -431.69 | 2.40 | 0.121 |
|  |  |  | scale(Plant richness site i):scale(Plant richness site j) | <0.01 | -0.06 | -434.09 | <0.01 | 0.949 |
|  |  | Random effects (obs = 351) | | Name | Variance | Std. Dev. |  |  |
|  |  |  | Site i (n = 26) | Intercept | 0.004 | 0.061 |  |  |
|  |  |  | Site j (n = 26) | Intercept | 0.005 | 0.074 |  |  |
|  |  | Fixed Effect - Seminatural LLS length | | Est. | z-value | AIC | LRT | p |
|  |  |  | No variables removed (null) | | | -432.82 |  |  |
|  |  |  | Intercept | 0.33 | 12.49 |  |  |  |
|  |  |  | scale(Semi-nat. LLS length) | -0.01 | -1.31 | -433.12 | 1.71 | 0.192 |
|  |  |  | Plant comp. similarity | 0.08 | 1.49 | -432.61 | 2.21 | 0.137 |
|  |  |  | scale(Plant richness site i):scale(Plant richness site j) | <0.01 | -0.06 | -434.82 | <0.01 | 0.953 |
|  |  | Random effects (obs = 351) | | Name | Variance | Std. Dev. |  |  |
|  |  |  | Site i (n = 26) | Intercept | 0.004 | 0.061 |  |  |
|  |  |  | Site j (n = 26) | Intercept | 0.005 | 0.073 |  |  |
|  |  | Fixed Effect - Forest edge length model | | Est. | z-value | AIC | LRT | p |
|  |  |  | No variables removed (null) | | | -432.69 |  |  |
|  |  |  | Intercept | 0.33 | 12.47 |  |  |  |
|  |  |  | scale(Forest edge length) | -0.01 | -1.26 | -433.12 | 1.57 | 0.210 |
|  |  |  | Plant comp. similarity | 0.08 | 1.49 | -432.47 | 2.22 | 0.136 |
|  |  |  | scale(Plant richness site i):scale(Plant richness site j) | <0.01 | -0.06 | -434.68 | <0.01 | 0.955 |
|  |  | Random effects (obs = 351) | | Name | Variance | Std. Dev. |  |  |
|  |  |  | Site i (n = 26) | Intercept | 0.004 | 0.061 |  |  |
|  |  |  | Site j (n = 26) | Intercept | 0.005 | 0.074 |  |  |
|  |  | Fixed Effect - Seminatural LLS cost model | | Est. | z-value | AIC | LRT | p |
|  |  |  | No variables removed (null) | | | -432.87 |  |  |
|  |  |  | Intercept | 0.33 | 12.48 |  |  |  |
|  |  |  | scale(Semi-nat. LLS cost) | -0.01 | -1.33 | -433.12 | 1.75 | 0.185 |
|  |  |  | Plant comp. similarity | 0.07 | 1.48 | -432.67 | 2.2 | 0.138 |
|  |  |  | scale(Plant richness site i):scale(Plant richness site j) | <0.01 | -0.06 | -434.87 | <0.01 | 0.956 |
|  |  | Random effects (obs = 351) | | Name | Variance | Std. Dev. |  |  |
|  |  |  | Site i (n = 26) | Intercept | 0.004 | 0.061 |  |  |
|  |  |  | Site j (n = 26) | Intercept | 0.005 | 0.074 |  |  |
|  |  | Fixed Effect - Forest edge cost model | | Est. | z-value | AIC | LRT | p |
|  |  |  | No variables removed (null) | | | -432.74 |  |  |
|  |  |  | Intercept | 0.33 | 12.47 |  |  |  |
|  |  |  | scale(Forest edge cost) | -0.01 | -1.28 | -433.12 | 1.63 | 0.202 |
|  |  |  | Plant comp. similarity | 0.07 | 1.48 | -432.55 | 2.2 | 0.138 |
|  |  |  | scale(Plant richness site i):scale(Plant richness site j) | <0.01 | -0.05 | -434.74 | <0.01 | 0.958 |
|  |  | Random effects (obs = 351) | | Name | Variance | Std. Dev. |  |  |
|  |  |  | Site i (n = 26) | Intercept | 0.004 | 0.061 |  |  |
|  |  |  | Site j (n = 26) | Intercept | 0.005 | 0.074 |  |  |

**Table S4**. T-family GLMM results testing solitary bee species compositional similarity between Danish site pairs across various distance measures (geographic, least-cost paths via seminatural LLS or forest edges). A null model without distance was included for comparison. The table shows likelihood ratio test statistics (LRT, AIC, p), estimated slopes (Est.), and effect sizes (z-values). Models control for plant species composition and richness differences via an interaction term. Intercept statistics are omitted.

| Danish sites: Solitary bees only | | | | | | | | |
| --- | --- | --- | --- | --- | --- | --- | --- | --- |
|  |  | Fixed Effect - null model | | Est. | z-value | AIC | LRT | p |
|  |  |  | No variables removed (null) | | | -423.75 |  |  |
|  |  |  | Intercept | 0.24 | 14.76 |  |  |  |
|  |  |  | Plant comp. similarity | 0.16 | 3.31 | -415.02 | 10.74 | 0.001 |
|  |  |  | scale(Plant richness _site i_):scale(Plant richness _site j_) | 0 | 0.23 | -425.7 | 0.05 | 0.817 |
|  |  | Random effects (obs = 351) | | Name | Variance | Std. Dev. |  |  |
|  |  |  | Site i (n = 26) | Intercept | <0.001 | <0.001 |  |  |
|  |  |  | Site j (n = 26) | Intercept | <0.001 | 0.022 |  |  |
|  |  |  | |  |  |  |  |  |
|  |  | Fixed Effect - Geographic distance model | | Est. | z-value | AIC | LRT | p |
|  |  |  | No variables removed (null) | | | -424.49 |  |  |
|  |  |  | Intercept | 0.24 | 14.95 |  |  |  |
|  |  |  | scale(Geographic distance) | -0.01 | -1.66 | -423.75 | 2.74 | 0.098 |
|  |  |  | Plant comp. similarity | 0.15 | 3.23 | -416.37 | 10.13 | 0.001 |
|  |  |  | scale(Plant richness _site i_):scale(Plant richness _site j_) | 0 | 0.24 | -426.44 | 0.06 | 0.814 |
|  |  | Random effects (obs = 351) | | Name | Variance | Std. Dev. |  |  |
|  |  |  | Site i (n = 26) | Intercept | <0.001 | <0.001 |  |  |
|  |  |  | Site j (n = 26) | Intercept | <0.001 | 0.021 |  |  |
|  |  |  | |  |  |  |  |  |
|  |  | Fixed Effect - Seminatural LLS length | | Est. | z-value | AIC | LRT | p |
|  |  |  | No variables removed (null) | | | -426.05 |  |  |
|  |  |  | Intercept | 0.24 | 15.1 |  |  |  |
|  |  |  | scale(Semi-nat. LLS length) | -0.01 | -2.08 | -423.75 | 4.3 | 0.038 |
|  |  |  | Plant comp. similarity | 0.15 | 3.19 | -418.26 | 9.79 | 0.002 |
|  |  |  | scale(Plant richness _site i_):scale(Plant richness _site j_) | 0 | 0.23 | -427.99 | 0.05 | 0.816 |
|  |  | Random effects (obs = 351) | | Name | Variance | Std. Dev. |  |  |
|  |  |  | Site i (n = 26) | Intercept | <0.001 | <0.001 |  |  |
|  |  |  | Site j (n = 26) | Intercept | <0.001 | 0.019 |  |  |
|  |  |  | |  |  |  |  |  |
|  |  | Fixed Effect - Forest edge length model | | Est. | z-value | AIC | LRT | p |
|  |  |  | No variables removed (null) | | | -426.8 |  |  |
|  |  |  | Intercept | 0.24 | 15.19 |  |  |  |
|  |  |  | scale(Forest edge length) | -0.01 | -2.26 | -423.75 | 5.05 | 0.025 |
|  |  |  | Plant comp. similarity | 0.15 | 3.14 | -419.27 | 9.53 | 0.002 |
|  |  |  | scale(Plant richness _site i_):scale(Plant richness _site j_) | <0.01 | 0.24 | -428.75 | 0.06 | 0.808 |
|  |  | Random effects (obs = 351) | | Name | Variance | Std. Dev. |  |  |
|  |  |  | Site i (n = 26) | Intercept | <0.001 | <0.001 |  |  |
|  |  |  | Site j (n = 26) | Intercept | <0.001 | 0.019 |  |  |
|  |  |  | |  |  |  |  |  |
|  |  | Fixed Effect - Semi-tural LLS cost model | | Est. | z-value | AIC | LRT | p |
|  |  |  | No variables removed (null) | | | -426.17 |  |  |
|  |  |  | Intercept | 0.24 | 15.07 |  |  |  |
|  |  |  | scale(Semi-nat. LLS cost) | -0.01 | -2.11 | -423.75 | 4.41 | 0.036 |
|  |  |  | Plant comp. similarity | 0.15 | 3.21 | -418.31 | 9.86 | 0.002 |
|  |  |  | scale(Plant richness _site i_):scale(Plant richness _site j_) | <0.01 | 0.23 | -428.11 | 0.05 | 0.817 |
|  |  | Random effects (obs = 351) | | Name | Variance | Std. Dev. |  |  |
|  |  |  | Site i (n = 26) | Intercept | <0.001 | <0.01 |  |  |
|  |  |  | Site j (n = 26) | Intercept | <0.001 | 0.02 |  |  |
|  |  |  | |  |  |  |  |  |
|  |  | Fixed Effect - Forest edge cost model | | Est. | z-value | AIC | LRT | p |
|  |  |  | No variables removed (null) | | | -426.96 |  |  |
|  |  |  | Intercept | 0.24 | 15.18 |  |  |  |
|  |  |  | scale(Forest edge cost) | -0.01 | -2.29 | -423.75 | 5.2 | 0.023 |
|  |  |  | Plant comp. similarity | 0.15 | 3.14 | -419.46 | 9.5 | 0.002 |
|  |  |  | scale(Plant richness _site i_):scale(Plant richness _site j_) | <0.01 | 0.25 | -428.9 | 0.06 | 0.804 |
|  |  | Random effects (obs = 351) | | Name | Variance | Std. Dev. |  |  |
|  |  |  | Site i (n = 26) | Intercept | 0 | 0 |  |  |
|  |  |  | Site j (n = 26) | Intercept | 0 | 0.019 |  |  |

**Table S5.** T-family GLMM results testing wild bee species compositional similarity between Norwegian site pairs across various distance measures (geographic, least-cost paths via seminatural LLS or forest edges). A null model without distance was included for comparison. The table shows likelihood ratio test statistics (LRT, AIC, p), estimated slopes (Est.), and effect sizes (z-values). Models control for plant species composition and richness differences via an interaction term. Intercept statistics are omitted.

| Norwegian sites: All wild bees | | | | | | | | |
| --- | --- | --- | --- | --- | --- | --- | --- | --- |
|  |  | Fixed Effect - null model | | Est. | z-value | AIC | LRT | p |
|  |  |  | No variables removed (null) | | | -557.75 |  |  |
|  |  |  | Intercept | 0.30 | 9.76 |  |  |  |
|  |  |  | Plant comp. similarity | 0.14 | 2.5 | -553.48 | 6.26 | 0.012 |
|  |  |  | scale(Plant richness _site i_):scale(Plant richness _site j_) | 0.03 | 3.95 | -544.75 | 15.00 | <0.001 |
|  |  | Random effects (obs = 493) | | Name | Variance | Std. Dev. |  |  |
|  |  |  | Site i (n = 40) | Intercept | 0.003 | 0.054 |  |  |
|  |  |  | Site j (n = 40) | Intercept | 0.004 | 0.067 |  |  |
|  |  |  | |  |  |  |  |  |
|  |  | Fixed Effect - Geographic distance model | | Est. | z-value | AIC | LRT | p |
|  |  |  | No variables removed (null) | | | -604.95 |  |  |
|  |  |  | Intercept | 0.30 | 10.22 |  |  |  |
|  |  |  | scale(Geographic distance) | -0.05 | -7.21 | -557.75 | 49.2 | <0.001 |
|  |  |  | Plant comp. similarity | 0.12 | 2.24 | -601.92 | 5.02 | 0.025 |
|  |  |  | scale(Plant richness _site i_):scale(Plant richness _site j_) | 0.01 | 1.86 | -603.51 | 3.44 | 0.064 |
|  |  | Random effects (obs = 493) | | Name | Variance | Std. Dev. |  |  |
|  |  |  | Site i (n = 40) | Intercept | 0.003 | 0.058 |  |  |
|  |  |  | Site j (n = 40) | Intercept | 0.004 | 0.065 |  |  |
|  |  |  | |  |  |  |  |  |
|  |  | Fixed Effect - Semi-tural LLS length | | Est. | z-value | AIC | LRT | p |
|  |  |  | No variables removed (null) | | | -608.16 |  |  |
|  |  |  | Intercept | 0.30 | 10.14 |  |  |  |
|  |  |  | scale(Semi-nat. LLS length) | -0.05 | -7.45 | -557.75 | 52.41 | <0.001 |
|  |  |  | Plant comp. similarity | 0.13 | 2.39 | -604.45 | 5.71 | 0.017 |
|  |  |  | scale(Plant richness _site i_):scale(Plant richness _site j_) | 0.01 | 1.88 | -606.65 | 3.51 | 0.061 |
|  |  | Random effects (obs = 493) | | Name | Variance | Std. Dev. |  |  |
|  |  |  | Site i (n = 40) | Intercept | 0.003 | 0.058 |  |  |
|  |  |  | Site j (n = 40) | Intercept | 0.004 | 0.063 |  |  |
|  |  |  | |  |  |  |  |  |
|  |  | Fixed Effect - Forest edge length model | | Est. | z-value | AIC | LRT | p |
|  |  |  | No variables removed (null) | | | -610.18 |  |  |
|  |  |  | Intercept | 0.30 | 10.25 |  |  |  |
|  |  |  | scale(Forest edge length) | -0.05 | -7.6 | -557.75 | 54.43 | <0.001 |
|  |  |  | Plant comp. similarity | 0.12 | 2.29 | -606.96 | 5.22 | 0.022 |
|  |  |  | scale(Plant richness _site i_):scale(Plant richness _site j_) | 0.01 | 2.02 | -608.14 | 4.04 | 0.044 |
|  |  | Random effects (obs = 493) | | Name | Variance | Std. Dev. |  |  |
|  |  |  | Site i (n = 40) | Intercept | 0.003 | 0.056 |  |  |
|  |  |  | Site j (n = 40) | Intercept | 0.004 | 0.064 |  |  |
|  |  |  | |  |  |  |  |  |
|  |  | Fixed Effect - Seminatural LLS cost model | | Est. | z-value | AIC | LRT | p |
|  |  |  | No variables removed (null) | | | -601.95 |  |  |
|  |  |  | Intercept | 0.29 | 9.98 |  |  |  |
|  |  |  | scale(Semi-nat. LLS cost) | -0.05 | -6.95 | -557.75 | 46.2 | <0.001 |
|  |  |  | Plant comp. similarity | 0.13 | 2.46 | -597.91 | 6.04 | 0.014 |
|  |  |  | scale(Plant richness _site i_):scale(Plant richness _site j_) | 0.01 | 2.1 | -599.59 | 4.36 | 0.037 |
|  |  | Random effects (obs = 493) | | Name | Variance | Std. Dev. |  |  |
|  |  |  | Site i (n = 40) | Intercept | 0.003 | 0.058 |  |  |
|  |  |  | Site j (n = 40) | Intercept | 0.004 | 0.064 |  |  |
|  |  |  | |  |  |  |  |  |
|  |  | Fixed Effect - Forest edge cost model | | Est. | z-value | AIC | LRT | p |
|  |  |  | No variables removed (null) | | | -609.37 |  |  |
|  |  |  | Intercept | 0.3 | 10.18 |  |  |  |
|  |  |  | scale(Forest edge cost) | -0.05 | -7.52 | -557.75 | 53.62 | <0.001 |
|  |  |  | Plant comp. similarity | 0.12 | 2.37 | -605.76 | 5.61 | 0.018 |
|  |  |  | scale(Plant richness _site i_):scale(Plant richness _site j_) | 0.01 | 2.24 | -606.4 | 4.97 | 0.026 |
|  |  | Random effects (obs = 493) | | Name | Variance | Std. Dev. |  |  |
|  |  |  | Site i (n = 40) | Intercept | 0.003 | 0.056 |  |  |
|  |  |  | Site j (n = 40) | Intercept | 0.004 | 0.063 |  |  |

**Table S6.** T-family GLMM results testing solitary bee species compositional similarity between Norwegian site pairs across various distance measures (geographic, least-cost paths via seminatural LLS or forest edges). A null model without distance was included for comparison. The table shows likelihood ratio test statistics (LRT, AIC, p), estimated slopes (Est.), and effect sizes (z-values). Models control for plant species composition and richness differences via an interaction term. Intercept statistics are omitted.

| Norwegian sites: Solitary bees only | | | | | | | | |
| --- | --- | --- | --- | --- | --- | --- | --- | --- |
|  |  | Fixed Effect - null model | | Est. | z-value | AIC | LRT | p |
|  |  |  | No variables removed (null) | | | -492.38 |  |  |
|  |  |  | Intercept | 0.35 | 12.71 |  |  |  |
|  |  |  | Plant comp. similarity | <0.01 | <0.01 | -494.38 | <0.01 | 1.000 |
|  |  |  | scale(Plant richness _site i_):scale(Plant richness _site j_) | 0.03 | 4.65 | -473.46 | 20.92 | <0.001 |
|  |  | Random effects (obs = 493) | | Name | Variance | Std. Dev. |  |  |
|  |  |  | Site i (n = 40) | Intercept | <0.001 | 0.014 |  |  |
|  |  |  | Site j (n = 40) | Intercept | 0.002 | 0.046 |  |  |
|  |  |  | |  |  |  |  |  |
|  |  | Fixed Effect - Geographic distance model | | Est. | z-value | AIC | LRT | p |
|  |  |  | No variables removed (null) | | | -513.4 |  |  |
|  |  |  | Intercept | 0.35 | 13.13 |  |  |  |
|  |  |  | scale(Geographic distance) | -0.03 | -4.85 | -492.38 | 23.02 | <0.001 |
|  |  |  | Plant comp. similarity | -0.02 | -0.32 | -515.3 | 0.10 | 0.746 |
|  |  |  | scale(Plant richness _site i_):scale(Plant richness _site j_) | 0.02 | 3.31 | -504.7 | 10.71 | 0.001 |
|  |  | Random effects (obs = 493) | | Name | Variance | Std. Dev. |  |  |
|  |  |  | Site i (n = 40) | Intercept | <0.001 | 0.016 |  |  |
|  |  |  | Site j (n = 40) | Intercept | 0.002 | 0.044 |  |  |
|  |  |  | |  |  |  |  |  |
|  |  | Fixed Effect - Seminatural LLS length | | Est. | z-value | AIC | LRT | p |
|  |  |  | No variables removed (null) | | | -516.77 |  |  |
|  |  |  | Intercept | 0.35 | 12.93 |  |  |  |
|  |  |  | scale(Semi-nat. LLS length) | -0.04 | -5.18 | -492.38 | 26.39 | <0.001 |
|  |  |  | Plant comp. similarity | -0.01 | -0.21 | -518.73 | 0.04 | 0.834 |
|  |  |  | scale(Plant richness _site i_):scale(Plant richness _site j_) | 0.02 | 3.27 | -508.33 | 10.45 | 0.001 |
|  |  | Random effects (obs = 493) | | Name | Variance | Std. Dev. |  |  |
|  |  |  | Site i (n = 40) | Intercept | <0.001 | 0.018 |  |  |
|  |  |  | Site j (n = 40) | Intercept | 0.002 | 0.045 |  |  |
|  |  |  | |  |  |  |  |  |
|  |  | Fixed Effect - Forest edge length model | | Est. | z-value | AIC | LRT | p |
|  |  |  | No variables removed (null) | | | -517.01 |  |  |
|  |  |  | Intercept | 0.35 | 13.11 |  |  |  |
|  |  |  | scale(Forest edge length) | -0.04 | -5.23 | -492.38 | 26.63 | <0.001 |
|  |  |  | Plant comp. similarity | -0.02 | -0.29 | -518.93 | 0.08 | 0.773 |
|  |  |  | scale(Plant richness _site i_):scale(Plant richness _site j_) | 0.02 | 3.39 | -507.81 | 11.21 | 0.001 |
|  |  | Random effects (obs = 493) | | Name | Variance | Std. Dev. |  |  |
|  |  |  | Site i (n = 40) | Intercept | <0.001 | 0.016 |  |  |
|  |  |  | Site j (n = 40) | Intercept | 0.002 | 0.044 |  |  |
|  |  |  | |  |  |  |  |  |
|  |  | Fixed Effect - Seminatural LLS cost model | | Est. | z-value | AIC | LRT | p |
|  |  |  | No variables removed (null) | | | -518.39 |  |  |
|  |  |  | Intercept | 0.35 | 12.88 |  |  |  |
|  |  |  | scale(Semi-nat. LLS cost) | -0.04 | -5.30 | -492.38 | 28.01 | <0.001 |
|  |  |  | Plant comp. similarity | -0.01 | -0.19 | -520.35 | 0.04 | 0.849 |
|  |  |  | scale(Plant richness _site i_):scale(Plant richness _site j_) | 0.02 | 3.43 | -508.91 | 11.48 | 0.001 |
|  |  | Random effects (obs = 493) | | Name | Variance | Std. Dev. |  |  |
|  |  |  | Site i (n = 40) | Intercept | <0.001 | 0.02 |  |  |
|  |  |  | Site j (n = 40) | Intercept | 0.002 | 0.044 |  |  |
|  |  |  | |  |  |  |  |  |
|  |  | Fixed Effect - Forest edge cost model | | Est. | z-value | AIC | LRT | p |
|  |  |  | No variables removed (null) | | | -520.01 |  |  |
|  |  |  | Intercept | 0.35 | 13.07 |  |  |  |
|  |  |  | scale(Forest edge cost) | -0.04 | -5.50 | -492.38 | 29.63 | <0.001 |
|  |  |  | Plant comp. similarity | -0.01 | -0.22 | -521.96 | 0.05 | 0.826 |
|  |  |  | scale(Plant richness _site i_):scale(Plant richness _site j_) | 0.02 | 3.55 | -509.75 | 12.26 | <0.001 |
|  |  | Random effects (obs = 493) | | Name | Variance | Std. Dev. |  |  |
|  |  |  | Site i (n = 40) | Intercept | <0.001 | 0.017 |  |  |
|  |  |  | Site j (n = 40) | Intercept | 0.002 | 0.043 |  |  |


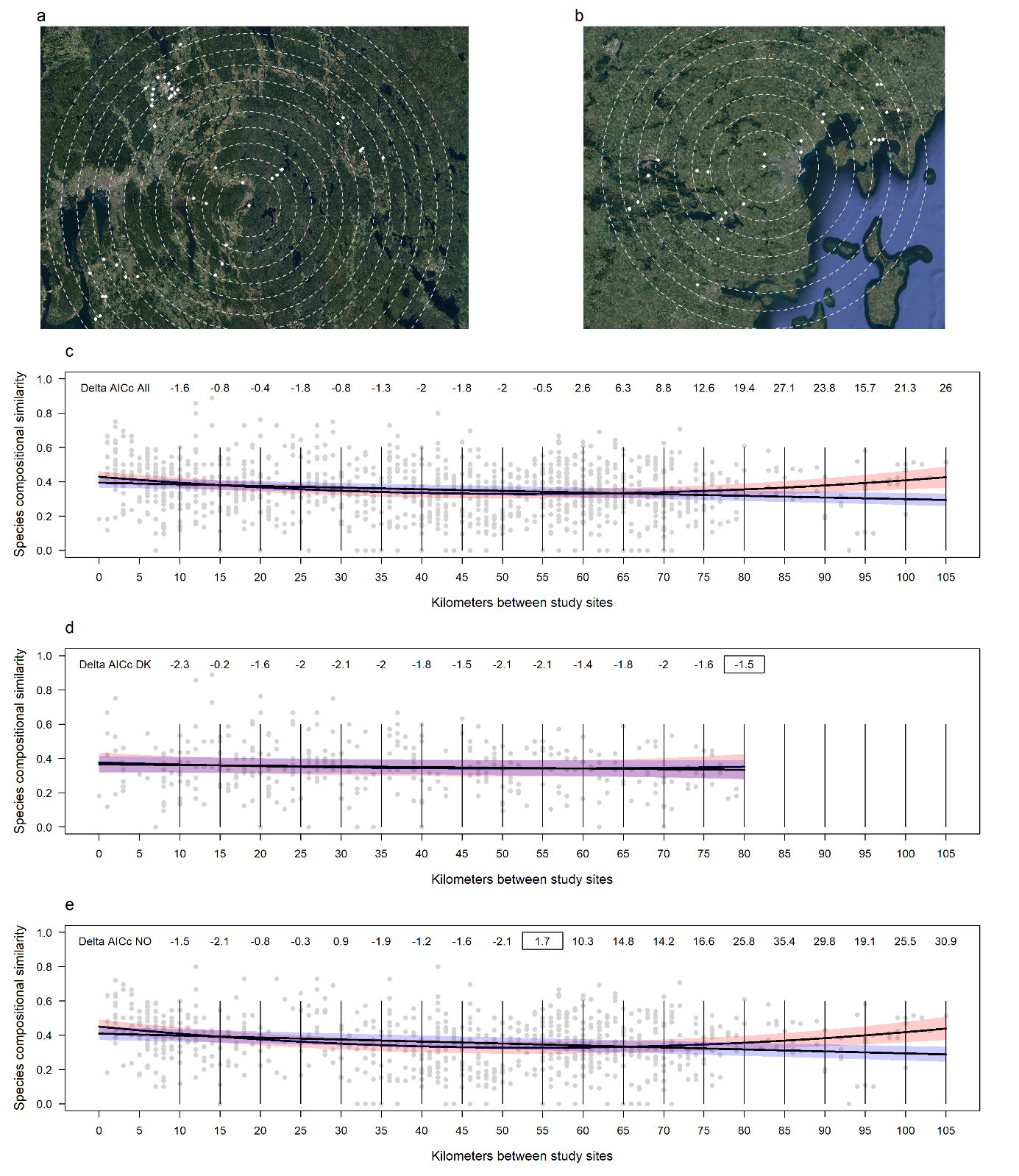


**Figure S1.** Study site locations in Norway (a) and Denmark (b), shown with 5 km distance buffers from the central site in each region. Species compositional similarity of bees between sites was modeled using Gaussian GLMMs with untransformed or polynomial distance terms, compared via AICc. Across all sites (c), similarity declined linearly up to 60 km, with polynomial models outperforming at larger distances (ΔAICc > 2). In Denmark (d), species compositional similarity showed no sign of a unimodal response across the range of site-pair distances. In Norway (e), a 55 km cutoff showed linear similarity decline (ΔAICc < 2). Regression lines (c-e) show model fits ± 2 SD for untransformed (blue) and polynomial (red) models.
